# Compensation of feature selection biases accompanied with improved predictive performance for binary classification by using a novel ensemble feature selection approach

**DOI:** 10.1186/s13040-016-0114-4

**Published:** 2016-11-18

**Authors:** Ursula Neumann, Mona Riemenschneider, Jan-Peter Sowa, Theodor Baars, Julia Kälsch, Ali Canbay, Dominik Heider

**Affiliations:** 1 Department of Bioinformatics, Straubing, 94315 Germany; 2 University of Applied Science, Weihenstephan-Triesdorf, Freising, 85354 Germany; 3 Wissenschaftszentrum Weihenstephan, Technische Universität München, Freising, 85354 Germany; 4 Department of Gastroenterology and Hepatology, University Hospital, University Duisburg-Essen, Essen, 45122 Germany; 5 Clinic for Cardiology, West German Heart and Vascular Centre Essen, University Hospital, University Duisburg-Essen, Essen, 45122 Germany

**Keywords:** Machine learning, Feature selection, Ensemble learning, Biomarker discovery, Random forest

## Abstract

**Motivation:**

Biomarker discovery methods are essential to identify a minimal subset of features (e.g., serum markers in predictive medicine) that are relevant to develop prediction models with high accuracy. By now, there exist diverse feature selection methods, which either are embedded, combined, or independent of predictive learning algorithms. Many preceding studies showed the defectiveness of single feature selection results, which cause difficulties for professionals in a variety of fields (e.g., medical practitioners) to analyze and interpret the obtained feature subsets. Whereas each of these methods is highly biased, an ensemble feature selection has the advantage to alleviate and compensate for such biases. Concerning the reliability, validity, and reproducibility of these methods, we examined eight different feature selection methods for binary classification datasets and developed an ensemble feature selection system.

**Results:**

By using an ensemble of feature selection methods, a quantification of the importance of the features could be obtained. The prediction models that have been trained on the selected features showed improved prediction performance.

**Electronic supplementary material:**

The online version of this article (doi:10.1186/s13040-016-0114-4) contains supplementary material, which is available to authorized users.

## Background

In the fields of predictive medicine as well as molecular diagnostics the need for simplification of datasets with many parameters frequently emerges. Therefore, approaches are necessary, which can identify important parameters (sometimes also referred to as features, independent variables, or predictor variables). Such quantifiable parameters that allow diagnostic validity are called biomarkers. In 2001, the Biomarkers Definitions Working Group of the American National Institute of Health defined a biomarker as “a characteristic that is objectively measured and evaluated as an indication of normal biologic processes, pathogenic processes, or pharmacologic responses to a therapeutic intervention“ [1]. Examples for biomarkers are serum parameters, genetic markers, or socio-demographic markers.

The detection of biomarkers can be conducted by computer-assisted approaches, namely feature selection (FS) methods. A great variety of FS techniques already exist. In general, these approaches can be separated into: filter methods, wrapper methods, and embedded methods. The first one is independent of any prediction model and therefore shows an advantage in regards of computation time compared to the other approaches. Filter methods use weighting measures, such as correlation coefficients [2] or mutual information [3]. The wrapper methods are computationally intensive, but in turn provide better accuracy compared to filter methods [4]. This type of approach occurs outside the model construction, however it uses the outcome as a guideline. The third type, the embedded methods, is an alternative to wrapper methods. These approaches combine the advantages of both methods stated above, namely the low computational costs and an adequate accuracy. This is due to the fact that the process of feature selection is already part of the model construction. There are three main criteria a feature selection method should meet, namely reliability, validity, and reproducibility. Methods that display these characteristics are called stable. Based on the definition of biomarkers, non-generalizable features are not considered to be reliable markers. There are several factors that can cause instability of the feature selection, e.g., the complexity of multiple biomarkers, a small-n-large-p-problem, or when the algorithm simply ignores stability [5, 6]. Thus, feature selection results have to be treated with care. For example, the Gini-index is widely used in predictive medicine, but it has also been demonstrated to deliver instable results due to unbalanced datasets [7, 8]. To counteract instability of feature selection methods, we developed an ensemble feature selection (EFS) method, which compensates biases of single FS. The idea of ensemble methods is already widely used in learning algorithms [9]. In this article we will introduce eight FS methods and our quantifying EFS method. We evaluated our EFS method compared to the state-of-the-art method AUC-FS with regard to the prediction performance in subsequent classification based on six different datasets. Furthermore, we compared the results with prediction models without pre-selection of features.

## Methods

With the development of the EFS method we take advantage of the benefits of multiple feature selection methods and combine their normalized outputs to a quantitative ensemble importance. The key features of our EFS method are:

1. The combination of widely known and extensively tested feature selection methods.
2. The balance of biases by using an ensemble.
3. The evaluation of EFS.

Eight different feature selection methods have been used for the EFS approach. Since random forests have drawn increased attention in the field of predictive medicine, four of the chosen feature selection methods are embedded in a random forest algorithm. Further, we considered the outcome of a logistic regression (i.e., the coefficients) as another embedded method as well as the filter methods median, Pearson-, and Spearman-correlation.

We used implementations in **R**(http://www.r-project.org/) for the different basal feature selection methods. Before we go into details a general data setting is introduced:Let vectors *X*_*i*_=(*x*_1,*i*_,…,*x*_*N*,*i*_) be the prediction variables for *i*∈{1,…*M*} and *Y*=(*y*_1_,…,*y*_*N*_) be the response variable. Altogether, a data matrix of size *N*×*M*+1 is received, where *N* is the number of samples and *M* is the number of prediction variables.

### Random forest

Random forests (RFs) are ensemble learning methods for classifications and regressions consisting of multiple decision trees [10]. RFs have been shown to give highly accurate predictions on biological [11–13] and biomedical data [14, 15]. There are different implementations of the RF algorithm in R available, which offer diverse feature selection methods. In the context of RFs, these feature selection methods are called variable importance measures (VIMs). We integrated two of the available implementations of RFs into our EFS method: (i) the RF method adapted from Breiman [10], which uses the CART (classification and regression tree) algorithm for individual node decisions, implemented in the R package *randomForest* and (ii) the cforest [16] implementation from the R-package *party*, because of its promising AUC score VIM. In RF approaches, randomness is gained by the general technique of bootstrap aggregating, also called bagging, meaning that for the tree building process only a subset of the data samples are chosen with replacement. We used 1000 decision trees in both RFs. In order to get robust results, we averaged the VIMs over 100 RF models.

The raw variable importance score for *X*_*i*_ is given by the average over the set of all decision trees *t*∈{1,…,*T*} in the RF:

$$\widehat{VI}_{X_{i}}= \frac{1}{T}\sum_{t=1}^{T} \widehat{VI}_{X_{i}}(t). $$

In addition, we define an indicator function *I*(*A*) by:

$$\begin{array}{@{}rcl@{}} I(A)= \left\{ \begin{array} {cl} 1, & \text{if the argument} A \text{is fulfilled,} \\ 0, & \text{otherwise.} \end{array}\right. \end{array} $$

#### Gini-index

The Gini-index is the sum of products between different class proportions over all classes for each variable, which is in the case of a binary classification:

$$G = 2p(1-p), $$

where $p=\frac {N_{1}}{N}$ is the proportion of one of the classes, in this case for response *Y*=1, and *N*_1_ is the number of units in this class.The Gini-index *G* defines a measure *d*_*ij*_ of the decrease in heterogeneity at node *j*:

$$d_{ij}= G - (\frac{N_{L}}{N}G_{L}+\frac{N_{R}}{N}G_{R}), $$

where *G*_*R*_ and *G*_*L*_ respectively are the Gini-indexes calculated for the following right and left nodes and *N*_*L*_ and *N*_*R*_ are the numbers of units in the left and right node after splitting. With this measure the variable importance for *X*_*i*_ in tree *t* is defined as:

$${VI}_{X_{i}}(t)=\sum_{j\in J}d_{ij}I(X_{i}\ splits\ at\ node\ j). $$

For deeper insights in the functionality of the Gini VIM we refer to [7].

#### Mean accuracy error-rate-based VIM

The mean accuracy error-rate-based VIM uses the out-of-bag (OOB) data. The OOB consists of the subset of all samples which are not used for the construction of decision trees: For each tree, the prediction error on the OOB portion of the data is recorded (error rates for classification, mean square errors for regression). This process is repeated after permuting each predictor variable. The difference between both is averaged over all trees, and normalized by the standard deviation of the differences, except the standard deviation is zero. For each tree *t*, we get the following formula:

$$\widehat{VI}_{X_{i}}(t) = \frac{1}{|B(t)|} \sum_{j\in B(t)}I(y_{j}=p_{j})-I(y_{j}=p_{j,\pi i}), $$

where *p*_*i*_ is the RF prediction of the response variable, *π* *i* is the permutation of the values in the *i*-th variable and *B*(*t*) is the OOB data for tree *t*.

#### Conditional error-rate-based VIM

In principle, the underlying mathematical model for the conditional error-rate-based VIM is the same as for the mean accuracy error-rate-based VIM. The conditional VIM takes biases in variable importance into account, which are generated by a correlation of the tested *X*_*i*_ with the other prediction variables.For *Z*=*X*_1_,…,*X*_*i*−1_,*X*_*i*+1_,…,*X*_*M*_ we calculate

$$\widehat{VI}_{X_{i}}(t) = \frac{1}{|B(t)|}\sum_{j\in B(t)} I(y_{j}=p_{j})-I(y_{j}=p_{j,\pi i|Z}), $$

where *B*(*t*) is the OOB data for tree *t*. In other words, the variable *X*_*i*_ is permuted, while *Z* is fixed at *Z*=*z*:=(*c* *p*_1_,…,*c* *p*_*i*−1_,*c* *p*_*i*+1_,…,*c* *p*_*M*_), consisting of the cut points for each variable in *Z*, which are defined through the partition of the feature space of *X*_*i*_ induced by the current tree *t*.

#### AUC-based VIM

In contrast to the aforementioned VIMs, the AUC-based VIM does not employ the error-rate, but instead uses the Area Under the Curve (AUC). It is calculated as the integral of the Receiver Operating Characteristic (ROC) curve, which is received by mapping the sensitivity against specificity for every possible cut-off between the two classes.

In contrast to error-rate-based methods, which give more weight to the majority class, the AUC does not favor any class. In previous studies the AUC was shown to be a particularly appropriate VIM for unbalanced data settings and should be considered as the state-of-the-art model [17, 18]. The AUC-score is an estimator for the probability that a randomly chosen sample from class *Y*=1 receives a higher class probability for class *Y*=1 than a randomly chosen sample from class *Y*=0. The variable importance for each tree *t* is calculated as:

$$\widehat{VI}_{X_{i}}(t) = {AUC}_{i}-{AUC}_{\pi i} $$

where *A* *U* *C*_*i*_ and *A* *U* *C*_*πi*_ respectively are the AUCs computed from the OOB observations in tree *t* before respectively after permuting the values of predictor *X*_*i*_.

### Logistic regression

Even though RFs have become very popular, it is not totally understood why the algorithm acts in its specific way. An embedded feature selection method, which is understood in more details, is the weighting system (i.e., coefficients) of the logistic regression. For feature selection, we access the model’s coefficients, i.e., the *β*−values of the regression equation. It should be noted that the range of features can strongly differ. Due to this fact, the *β*-coefficients of parameters are not directly comparable. To provide comparability of the variables’ importances, we conducted a z-transformation:

$$z_{X}=\frac{X-\overline{X}}{s_{X}}, $$

where $\overline {X}$ is the mean and *s*_*X*_ the standard deviation of variable *X*, respectively. Through standardization by z-transformation, the mean of *β*-coefficients become zero with a standard deviation of 1, thus assuring that the features all have the same domain. Subsequently, the values are ordered according to their absolute values in decreasing order.

### Correlation coefficient

The correlation between any two features can be described as the quantification of the extent of statistical dependence between them, which can be quantified by different correlation coefficients. We used the approach of [19] to select features that are highly correlated with the dependent variable, but show only low correlation with other predictors. We used a threshold for the correlation between the predictor variables of *p*=0.7. In order to avoid collinearity a threshold of 0.7 is most frequently used [20], although recommendations for more restrictive (e.g., 0.4 [21]) and less restrictive (e.g., 0.8 [22]) thresholds exist. In our study, we adopted two correlation coefficients, namely the Pearson product-moment correlation and the Spearman rank correlation coefficient.

#### Pearson

For any two features *X* and *Y* with samples *j*=1,…,*n*, the Pearson product-moment correlation coefficient is defined as

$$ r_{XY}=\frac{\sum_{j=1}^{n}(x_{j}-\overline{x})(y_{j}-\overline{y})}{\sqrt{\sum_{j=1}^{n}(x_{j}-\overline{x})^{2} \sum_{j=1}^{n}(y_{j}-\overline{y})^{2}} }, $$

where $\overline {X}$ and $\overline {y}$ are the sample means of *X* and *Y*.

#### Spearman

For the Spearman rank correlation coefficient we observe the sample’s ranks *r* *k*(*x*_*i*_) and *r* *k*(*y*_*i*_) of the features *X*=(*x*_1_,…,*x*_*n*_) and *Y*=(*y*_1_…,*y*_*n*_) and compute

$$\rho =1-6 \sum_{j=1}^{n}\frac{{d_{i}^{2}}}{n(n^{2}-1)}, $$

where *d*_*i*_=*r* *k*(*x*_*i*_)−*r* *k*(*y*_*i*_).

### Median

For the median feature selection, we used a Mann-Whitney-U test [23] comparing the positive and negative class of the response variable *Y*. The test evaluates following hypothesis: Since *m* *e* *d*_0_ and *m* *e* *d*_1_ are the medians of the negative and positive class of a predictor variable, the null hypothesis for each predictor variable is defined as:

$$H_{0}: {med}_{0}={med}_{1}. $$

The resulting p-values of the Mann-Whitney-U test are used as scoring system for the feature selection. Thus, a smaller p-value indicates a higher importance.

### Ensemble feature selection

Feature selection methods as a preprocessing step for supervised learning algorithms provide several benefits, such as reduced computational costs (e.g., training time, storage requirements), but also improved prediction performance. However, different feature selection methods provide different subsets of features. Hence they give rise to sample selection bias. In general, the aim of supervised learning algorithms is to find a suitable hypothesis which makes the best prediction for a particular problem. Improvements can be achieved by combining multiple hypotheses instead of testing only one. This is the main concept of ensemble learning methods. Ensemble techniques are widely used in machine learning algorithms to achieve higher stability. The RF algorithm is an example for bootstrap aggregating [24]. This technique combines several prediction models using a randomly drawn subset of the training data. Another type of ensemble learning methods are boosting algorithms, which merge several weak classifiers to a stronger one. The most popular implementation is AdaBoost [25].

In the current study, we developed a stable feature selection procedure, which is based on the idea of ensemble learning. For our EFS method we integrated eight different feature selection methods and normalized all individual outputs to a common scale, an interval from 0 to 1. Thereby we ensure the comparability between different FS methods and conserve the distances of importance between one feature to another. This normalization is achieved in two different ways: For all feature selections, except for the median, the absolute value of the FS method output is a value which illustrates the increase of importance. By dividing through the maximum value we get values between 1 and 0:

$${imp}_{X_{i}}=\frac{\beta_{i}}{\max(\beta_{m})_{m\in M}}. $$

In the case of the median FS we receive a *p*-value for each feature *X*_*i*_, which is normalized as follows:

$${imp}_{X_{i}} = 1-p_{i}+\min(p_{i}). $$

For the four RF based VIMs, we computed 100 repetitions and averaged the importance for each feature. This procedure guarantees a higher robustness of the feature importance and the selected subset.

We evaluated the selected subsets by using a logistic regression model with a leave-one-out cross validation (LOOCV) to avoid overfitting. LOOCV is known to give inflated variance estimation [26], but in our study we used the LOOCV only for comparing the different methodologies. The EFS system selects those parameter that have a higher importance than the mean importance:

$${imp}_{X_{i}}>\overline{imp_{X_{M}}}, $$

where $\overline {imp_{X_{M}}}$ symbolizes the mean of all variable importances. Alternatively, the median or Q3 could be used as well, however, both would lead to a fixed number of selected parameter irrespective of their relevance for the subsequent classification model.

The logistic regression model based on the EFS-selected features was then compared to logistic regression models either trained on all features and on features selected by the AUC-based VIM, which is considered to be one of the state-of-the-art methods for feature selection. We examined the AUC-values of the ROC curves with ROCR [27]. Additionally, the improvement in performance between the AUC-based VIM, the EFS subset, and the model without feature selection is measured by a comparison of the AUCs via the method of DeLong et al. [28].

### Datasets

To evaluate our EFS method, we used six different datasets. An overview of the datasets is given in Table 1.

**Table 1:** Overview of datasets. Number of features after removing samples with missing values

Dataset	No. of Samples	No. of Features	Categorical	Numeric
MI-Mortality	406	14	7	7
Fibrosis	101	26	7	19
FLIP	103	13	6	7
SPECTF	267	44	44	0
Sonar	208	60	0	60
WBC	569	30	0	30

The first dataset *MI-Mortality* was provided by the Clinic for Cardiology, West German Heart and Vascular Centre Essen of the University Hospital Duisburg-Essen. It consists of 14 socio-demographic and serum parameters from 406 patients. The purpose of this study was to examine which parameters are important for the mortality prediction after treatment on myocardial infarction. The data was collected during a follow-up study of [29].

The Department of Gastroenterology and Hepatology of the University Hospital Duisburg-Essen provided the datasets *Fibrosis* [30] and *FLIP*, which again consist of socio-demographic and serum parameters. Both deal with different scores to predict fibrosis.

*SPECTF* is a dataset from the UCI Machine Learning Repository [31]. It describes diagnosing of cardiac Single Proton Emission Computed Tomography (SPECT) images. The class-variable is distinguishing between normal (=0) and abnormal (=1).

The *Sonar* dataset has also been retrieved from the UCI Machine Learning Repository and obtained by bouncing sonar signals off a metal cylinder or rock at various angles and under various conditions. The prediction model should be able to distinguish between rocks and metal cylinders.

In the *WBC* dataset a classification between benign and malignant tumors in breast cancer samples is intended. Benign tumors are not cancerous, thus these samples are class 0. Malignant tumor samples are class 1.

In order to reduce the number of missing values in the datasets, features with more than 20% missing values were discarded. Additionally, columns with zero variance were removed.

## Results

### Selected features

The number of selected features from EFS and AUC-FS varies for each dataset. The Gini FS method is known to prefer categorical variables with many categories and disregards potential important binary prediction variables [32]. In contrast to the Gini FS, we could observe that the variable type did not play a decisive role for the importance. Through aggregating different FS methods into an ensemble, biases of individual methods are compensated.

**Fig. 1:**
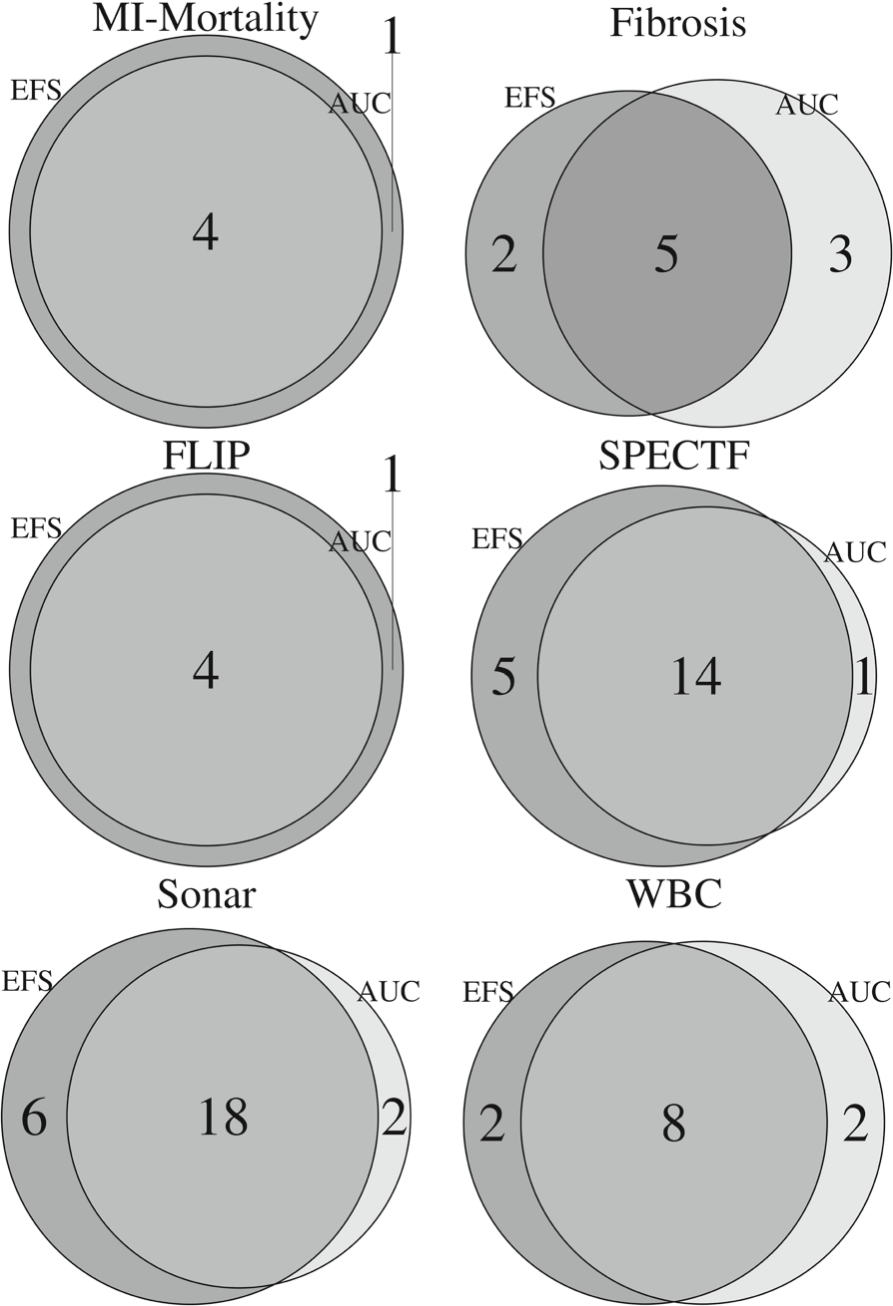
Venn diagrams. Comparison of feature subsets retrieved from AUC importance and EFS importance

In Fig. 1 Venn diagrams are shown, illustrating the feature subsets derived from the AUC-FS and EFS, respectively. The Venn diagrams show no distinct trend for the number of features that were selected by the respective method, i.e., in some datasets EFS selects more features than the AUC-FS, while in other datasets it is the other way around.

For the *Fibrosis* data the selected subset of AUC-FS contains eight features, whereas the EFS subset consists of only seven. Five features have been selected by both methods, while the other features are disjoint. The *WBC* dataset yielded a similar result. Both methods selected a subset of ten features, with eight features being selected by both methods. The results of the *MI-Mortality* data and *FLIP* data are similar: EFS selected a subset of five features while AUC-FS returned four features, which all are contained in the EFS selected subset. The datasets of the *SPECTF* resp. *Sonar* studies also deliver analogous subset schemes. The major part of selected features are chosen by both FS methods (14 and 18, respectively). Our EFS method considered five and six additional features, while the AUC-FS selected one and two additional features, which do not occur in the intersection of both subsets.

The EFS selected more features than the AUC-FS in four out of six cases, however the percentages of selected features out of all possible prediction variables ranged from 26.9 to 43.2% (cf. Table 2).

**Table 2:** Types of selected features. Evaluation of the selected features subsets of AUC-FS and EFS

Dataset	AUC-FS selected	EFS selected	EFS/all in %	Numeric*	Categorical*
MI-Mortality	4	5	35.7	3	2
Fibrosis	8	7	26.9	5	3
FLIP	4	5	38.5	3	2
SPECTF	15	19	43.2	0	19
Sonar	20	24	40.0	24	0
WBC	10	10	33.3	9	1

^*^refers to the EFS selected features

### Performance evaluation

**Fig. 2:**
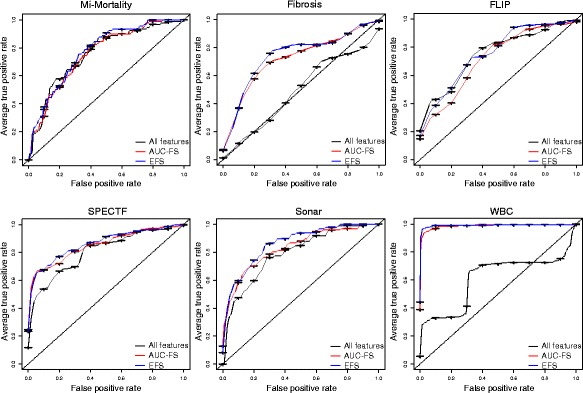
Performance of logistic regression models. On the y-axis the sensitivity and on the x-axis the specificity is shown. Three ROC curves are shown per dataset: of all features (*solid*), the AUC-FS selected (*dashed*) and the EFS selected (*twodashed*) features. The *dotted line* marks the performance of random guessing

In order to evaluate our EFS method in comparison to the AUC-FS, we used a logistic regression model with LOOCV. Additionally, we trained a logistic regression model without feature selection. Table 3 summarizes the results for all datasets. The resulting ROC curves are shown in Fig. 2.

**Table 3:** Results on datasets

Dataset	All [CI]	AUC-FS [CI]	EFS [CI]	AUC-FS vs. EFS*	all vs. EFS**
MI-Mortality	0.758 [0.700, 0.800]	0.757 [0.704, 0.811]	0.776 [0.725, 0.826]	0.228	0.201
Fibrosis	0.493 [0.300, 0.600]	0.681 [0.537, 0.824]	0.746 [0.617, 0.874]	0.273	**0.018**
FLIP	0.759 [0.600, 0.900]	0.723 [0.582, 0.863]	0.761 [0.633, 0.890]	0.254	0.971
SPECTF	0.807 [0.700, 0.900]	0.856 [0.811, 0.901]	0.865 [0.821, 0.910]	0.444	4.68e-4
Sonar	0.792 [0.700, 0.900]	0.840 [0.787, 0.894]	0.862 [0.813, 0.911]	0.200	**0.009**
WBC	0.611 [0.600, 0.700]	0.987 [0.977, 0.998]	0.991 [0.981, 1.000]	**0.020**	**1.21e-41**

Column 1 to 3 are AUCs values of all features, selected by AUC-FS and by the EFS with confidential intervalls in brackets. The last two columns show the *p*-values of the comparison by the method of [28]. The function compares the AUC of the ROC curves of (*) the AUC-FS and EFS method and (**) of all parameters and EFS outcome. Statistical significant *p*-values are printed in bold

For each dataset, the resulting model trained on the EFS selected subset of features performed superior compared to the models trained either on the AUC-FS selected features or on all features without selection.

However, the EFS showed a significantly higher AUC value only for the dataset WBC. For all other datasets, the AUCs were higher for the EFS compared to the AUC-FS as well, however the results were not significant: MI-Mortality (*p* = 0.228), Fibrosis (*p* = 0.273), FLIP (*p* = 0.254), SPECTF (*p* = 0.444), Sonar (*p* = 0.2), and WBC (*p* = 0.02).

The model using the EFS selected features showed significant higher AUC values compared to the model trained without feature selection for all datasets except MI-Mortality and FLIP (*p* = 0.201 and *p* = 0.971, respectively). Taken together, throughout all datasets we can observe an enhancement of performance by using the EFS method, although it is not significant in all datasets.

Additionally, we evaluated the robustness of our EFS approach by using permutation tests [33, 34]. To this end, the logistic regression models are compared to models that are trained on randomly permuted class labels. P-values for all datasets were less than 0.001.

Moreover, we evaluated the stability of the EFS approach in terms of selected features. To this end, we evaluated the variance of the importance of the five most important features using a 10-fold cross-validation of the datasets repeated 10 times. Furthermore, we used the Jaccard-index [35] as a stability score, described by the following formula:

$$ J(S_{1},\ldots,S_{n})=\frac{|S_{1}\cap\ldots\cap S_{n}|}{|S_{1}\cup\ldots\cup S_{n}|}, $$

where *S*_1_,…,*S*_*n*_ are different subsets of features. Thereby, a Jaccard-index close to 1 represents a high similarity of feature subsets. It turned out that EFS gives highly stable results with variances of the importance values less than 0.0235. Moreover, the Jaccard-index of the selected features by EFS was 1 for all data sets. Table 4 shows all variances of the importance and the corresponding boxplots can be found in the Additional file 1.

**Table 4:** Variance of feature importances. Variance of the five most important features of a 10-fold cross-validation

Dataset	Variance #1	Variance #2	Variance #3	Variance #4	Variance #5
MI-Mortality	0.001759124	0.004694053	0.004904828	0.003720571	0.001580310
Fibrosis	0.003124527	0.008085472	0.019901386	0.009202372	0.019804508
FLIP	0.006604973	0.011325453	0.014731007	0.023499884	0.020140657
SPECTF	0.000380482	0.014946809	0.011520607	0.005807655	0.002880478
Sonar	0.003887830	0.001792209	0.003004598	0.003115140	0.002680274
WBC	0.001071784	0.001769331	0.002912278	0.000387555	0.001096465

## Discussion

Feature selection methods have been studied for several decades (e.g., [36]). There are already many publications [37–41] on how to improve the performance of feature selection methods.

We provide an ensemble feature selection tool to conduct a feature selection for binary classification, which showed promising performance on all datasets. In contrast to ensemble methods of previous studies [42–44], the aim of this work was to combine filter and embedded methods. Due to their focus on predictions, embedded methods usually attain a higher prediction performance, whereas the advantage of filter methods are low computational cost and low complexity. By using ensembles, the advantages of both strategies can be combined and individual biases are alleviated. Concerning the enhanced approximation of embedded methods, we excluded wrapper methods from our study.

The cforest method requires more time than any other component of the EFS algorithm, thus calculations of datasets with hundreds of thousands of features would take up a lot of CPU time. A workload saving alternative would be a reduction of the repetition rate of the RF algorithms, in particular of the cforest algorithms. However, in turn this will negatively affect the VIM’s robustness. In our computations the repetition rate was set to 100 and the average variable importance was reported. Since, there is no generalization on how many repeats are necessary to get a robust result.

The evaluation of feature subsets depicted in the Venn diagrams reflects that in four out of six cases our EFS method selects more features than the AUC-FS. We assume that the reason for this phenomenon is based on the importance weighting system of the AUC-FS. As threshold for the decision which features are considered to be the most important ones, the respective mean over all importance values was taken. If there are only a few features lying above average, this might be an indication that the values of those features which are considered important are overestimated compared to the non-selected features. Thus the mean increases and less features reach that threshold. Alternatively, the opposite case could be true, meaning in one or more of the other feature selection methods the assigned importance values hardly differ. This in turn has an alleviating factor on the importance values of our ensemble of feature selection methods.

In the current study, we used the logistic regression method to analyze the performance of our EFS. For binary classification, logistic regression is the statistical method of choice, in particular in the field of predictive medicine [45]. It has the ability of detecting possible causal relationships between features. By conducting a z-transformation on the whole dataset the relationships become easy to interpret via the *β*-coefficients. Although the logistic regression model has many advantages, the prediction performance could be improved by using other predictive models in future studies. To get a broader and more generalizable rating for the results of our EFS method, an evaluation by methods such as support vector machines or RFs could additionally be conducted.

The output of all individual feature selection methods is normalized and summed up to our EFS result using the same weighting for all methods. However, there are more possibilities how the ensemble importance of features can be calculated, such as majority vote or by a weighting system. A weighting system could consider the individual robustness of each FS method, whereas a majority vote does not provide comparability between the importance of features. This issue could be solved by a weighted majority vote. For more details on fusion methods we refer to [9].

We determined several thresholds for the computation, namely the number of repetitions of the RF algorithms (100 times), the threshold of missing values (20%), and the correlation threshold between the dependent variables (0.7). In some data cases varying these thresholds might yield a better performance. However, for comparability reasons we used fixed thresholds for all datasets.

We also examined the subsets of features selected by the EFS method without the AUC-FS to estimate the influence of the AUC-FS. The selected features are essentially the same (cf. Table 5). In three datasets the subsets are slightly larger, which supports our theory on the overestimating effect of the AUC-FS on relevant feature’s importance.

**Table 5:** Quantity of selected features. Number of selected features of our EFS method with and without the AUC-FS

Dataset	EFS	EFS without AUC-FS	Intersection
MI-Mortality	5	5	5
Fibrosis	7	9	7
FLIP	5	5	5
SPECTF	19	20	19
Sonar	24	24	24
WBC	10	11	9

By the stability-test we proofed, that the EFS method is a stable and reliable approach for binary classification.

## Conclusion

In the current study, we could show the advantages of our EFS method for binary classification data, namely the robustness and stability of feature ranking and subset selection. The evaluation of prediction performance via ROC curves of a logistic regression model showed an improvement of the prediction based on the EFS selected features compared to all features on every tested dataset.

Further investigations on the topic of enhancing feature selection methods will be conducted in future. Firstly, we will evaluate our EFS method on high-dimensional data, such as data retrieved from microarray or next-generation sequencing analyses. So far we used datasets with less than 600 samples and a maximum of 60 features. Secondly, in future studies we would like to investigate how our method deals with multiple classes instead of binary classification. Therefore, it will be necessary to substitute the median feature selection method with an appropriate alternative. Another interesting application will be the extension on regression models where classes are replaced by continuous values. Another direction of our future work on EFS methods will concern the composition of our FS method set. By combining feature selection algorithms the accuracy will improve by the expense of increased complexity. Using an ensemble of several simple methods can gain a higher accuracy than one complex method (cf. [9]). Due to this theory, an evaluation is needed on which FS methods are mandatory to gain a maximum accuracy.

## Additional file

Additional file 1 Boxplots of five most important features in bootstrapping analyses. (JPEG 199 kb)

## Abbrevations

AUC: Area under the curve
CART: Classification and regression tree
CPU: Central processing unit
EFS: Ensemble feature selection
FS: Feature selection
LOOCV: Leave-one-out cross validation
OOB: Out-of-bag
RF: Random forest
ROC: Receiver operating characteristic
VIM: Variable importance measure
