# Supplementary figures and images for: Compensation of feature selection biases accompanied with improved predictive performance for binary classification by using a novel ensemble feature selection approach
Source: BioData Min. 2016 Nov 18;9:36. doi: 10.1186/s13040-016-0114-4 (PMC5116216; doi:10.1186/s13040-016-0114-4)

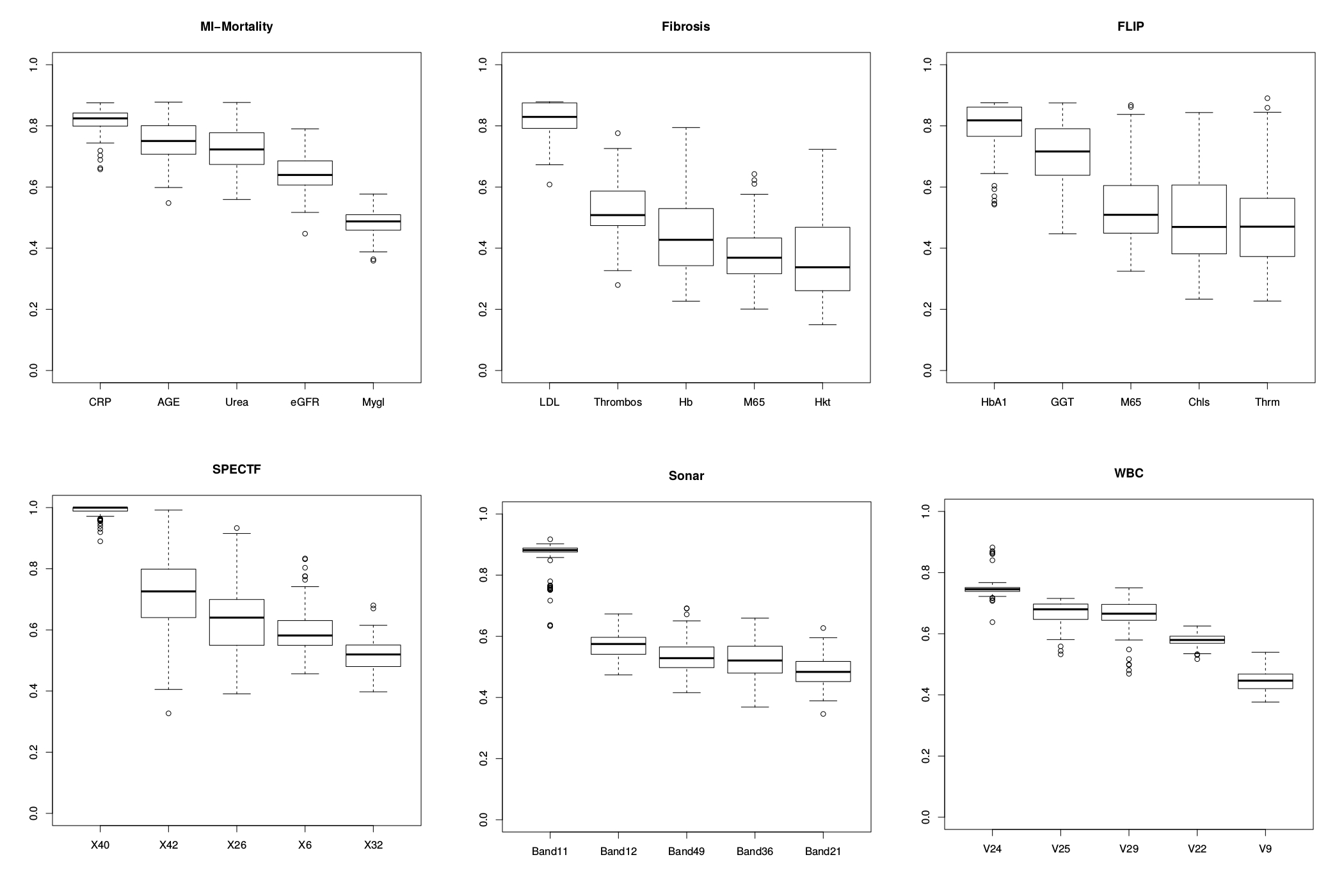

Supplement: Additional file 1 — Boxplots of five most important features in bootstrapping analyses. (JPEG 199 kb) [file 13040_2016_114_MOESM1_ESM.jpeg]
